# People Bouncing on Trampolines: Dramatic Energy Transfer, a Table-Top Demonstration, Complex Dynamics and a Zero Sum Game

**DOI:** 10.1371/journal.pone.0078645

**Published:** 2013-11-13

**Authors:** Manoj Srinivasan, Yang Wang, Alison Sheets

**Affiliations:** Mechanical and Aerospace Engineering, The Ohio State University, Columbus, Ohio, United States of America; Rensselaer Polytechnic Institute, United States of America

## Abstract

Jumping on trampolines is a popular backyard recreation. In some trampoline games (e.g., “seat drop war”), when two people land on the trampoline with only a small time-lag, one person bounces much higher than the other, as if energy has been transferred from one to the other. First, we illustrate this energy-transfer in a table-top demonstration, consisting of two balls dropped onto a mini-trampoline, landing almost simultaneously, sometimes resulting in one ball bouncing much higher than the other. Next, using a simple mathematical model of two masses bouncing passively on a massless trampoline with no dissipation, we show that with specific landing conditions, it is possible to transfer all the kinetic energy of one mass to the other through the trampoline – in a single bounce. For human-like parameters, starting with equal energy, the energy transfer is maximal when one person lands approximately when the other is at the bottom of her bounce. The energy transfer persists even for very stiff surfaces. The energy-conservative mathematical model exhibits complex non-periodic long-term motions. To complement this passive bouncing model, we also performed a game-theoretic analysis, appropriate when both players are acting strategically to steal the other player's energy. We consider a zero-sum game in which each player's goal is to gain the other player's kinetic energy during a single bounce, by extending her leg during flight. For high initial energy and a symmetric situation, the best strategy for both subjects (minimax strategy and Nash equilibrium) is to use the shortest available leg length and not extend their legs. On the other hand, an asymmetry in initial heights allows the player with more energy to gain even more energy in the next bounce. Thus synchronous bouncing unstable is unstable both for passive bouncing and when leg lengths are controlled as in game-theoretic equilibria.

## Introduction

**Figure 1:**
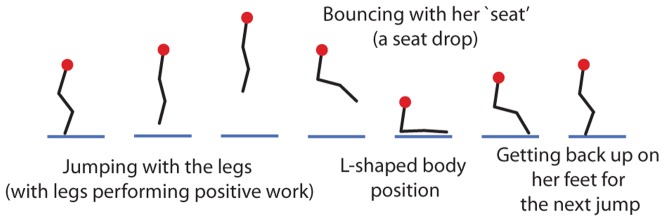
A two-person game on a trampoline: Seat drop war. Only one player shown. Each player alternatively bounces with her feet and her ‘seat’. The sequence of body configurations for one player is shown schematically. The other player goes through a similar sequence of configurations, but possibly with a phase difference.

Bouncing on a trampoline has evolved from a backyard activity for children to an Olympic Sport. While Olympic trampolining only has one person bouncing on a trampoline, in its recreational form, it is quite common for more than one person to bounce on the trampoline simultaneously. In particular, children play a two-person game on trampolines called “seat drop war.” In this game, each player bounces alternatively with her feet and her ‘seat’ (being in an L-shaped body configuration), as shown in Figure 1. See also movie S1, showing this game being played. Each player is able to increase her mechanical energy while bouncing (jumping) with her feet by performing mechanical work with her legs, but she essentially bounces passively when bouncing on her seat (and probably loses some energy due to damping). The goal of this game is to be the last player bouncing. As the game progresses with the two players bouncing alternatively with their feet and their seat, the relative phasing between their bounces typically changes: sometimes the players bounce out of phase and sometimes they bounce in phase. The game often ends with one person having so little upward velocity when bouncing on her seat that she is unable to get back on her feet for the next bounce. Often, associated with this loss, the second player appears to have gained most of the energy lost by the first player, thereby bouncing higher than usual. This article is motivated by this apparently dramatic energy transfer between the players, which typically happens during a bounce in which the two players are simultaneously in contact with trampoline for some overlapping time period.

Here, we show that the dramatic energy transfer is observed even in the passive bouncing of inanimate masses. We first describe a simple physical demonstration of the energy transfer: dropping two balls simultaneously onto a small trampoline sometimes results in one ball bouncing much higher than the other. Then, we construct a simple energy-conservative mathematical model, with the two people modeled as masses bouncing passively on a trampoline. This model also exhibits the dramatic energy transfer observed in seat drop war. We call the energy transfer ‘dramatic’ because essentially all the energy of one person/ball gets transferred to the other in a single brief interaction. We show that there is typically an optimal difference between the landing times of the two masses (hereafter called the ‘contact time-lag’) that maximizes energy transfer. The mathematical model, in absence of dissipation or sideways movement of masses, displays complex non-periodic motion, with repeated transfer of energy between the two masses.

Finally, we make a first step at analyzing the game, not as a simple passive dynamics problem involving two balls bouncing, but as a strategic competitive game between two players from a game theoretic perspective, obtaining the optimal strategies for the two players for the zero-sum game.

## Results

### A physical demonstration: Two balls on a trampoline

To illustrate that energy transfer between people on a trampoline can happen through purely passive mechanisms, we designed a simple table-top demonstration involving a store-bought mini-trampoline and two balls (see also Materials and Methods).

**Figure 2:**
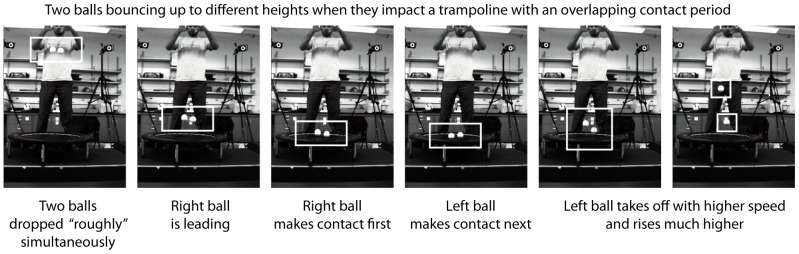
A table-top demonstration. This figure shows a sequence of frames illustrating two balls dropped almost simultaneously onto a mini-trampoline, bouncing back up to very different heights. We see that the ball that makes contact second, rises much higher, as also seen in the mathematical models. See slow motion video in movie S2.

Figure 2 shows a series of key frames, illustrating the energy transfer between the two (tennis) balls, dropped nearly but not exactly simultaneously. The two balls contact the trampoline at slightly different times, with some overlapping period when they are both in contact with the trampoline. The mass that makes contact with the trampoline second bounces much higher. See also movie S2, which shows this specific example in slow motion, and also other examples illustrating how when the masses make contact with the trampoline approximately simultaneously, they bounce up to about the same height.

We did not perform carefully controlled drops, make detailed measurements of the resulting bounces, or try to make this table-top demonstration a dynamically scaled version of two humans bouncing on a larger trampoline. We intend this only as a demonstration of the phenomenon.

When dropped by human hands, the two balls often land at slightly different times due to human motor variability, resulting in different amounts of overlap between their contact phases with the trampoline. As a consequence, as seen from the mathematical models below, the rise heights of the masses after the bounce have corresponding variability. When there is no contact overlap, as happens often (if the drops are not nearly simultaneous), there is no dramatic energy transfer.

### A mathematical model: passive bouncing of two masses

When people bounce on trampolines, they perform positive mechanical work with their legs to counteract any loss of energy (through passive dissipation or active negative leg work). Here, for simplicity, we restrict ourselves to energy-conservative models: no leg work or dissipation. See Materials and Methods for simulation details for these mathematical models.

**Figure 3:**
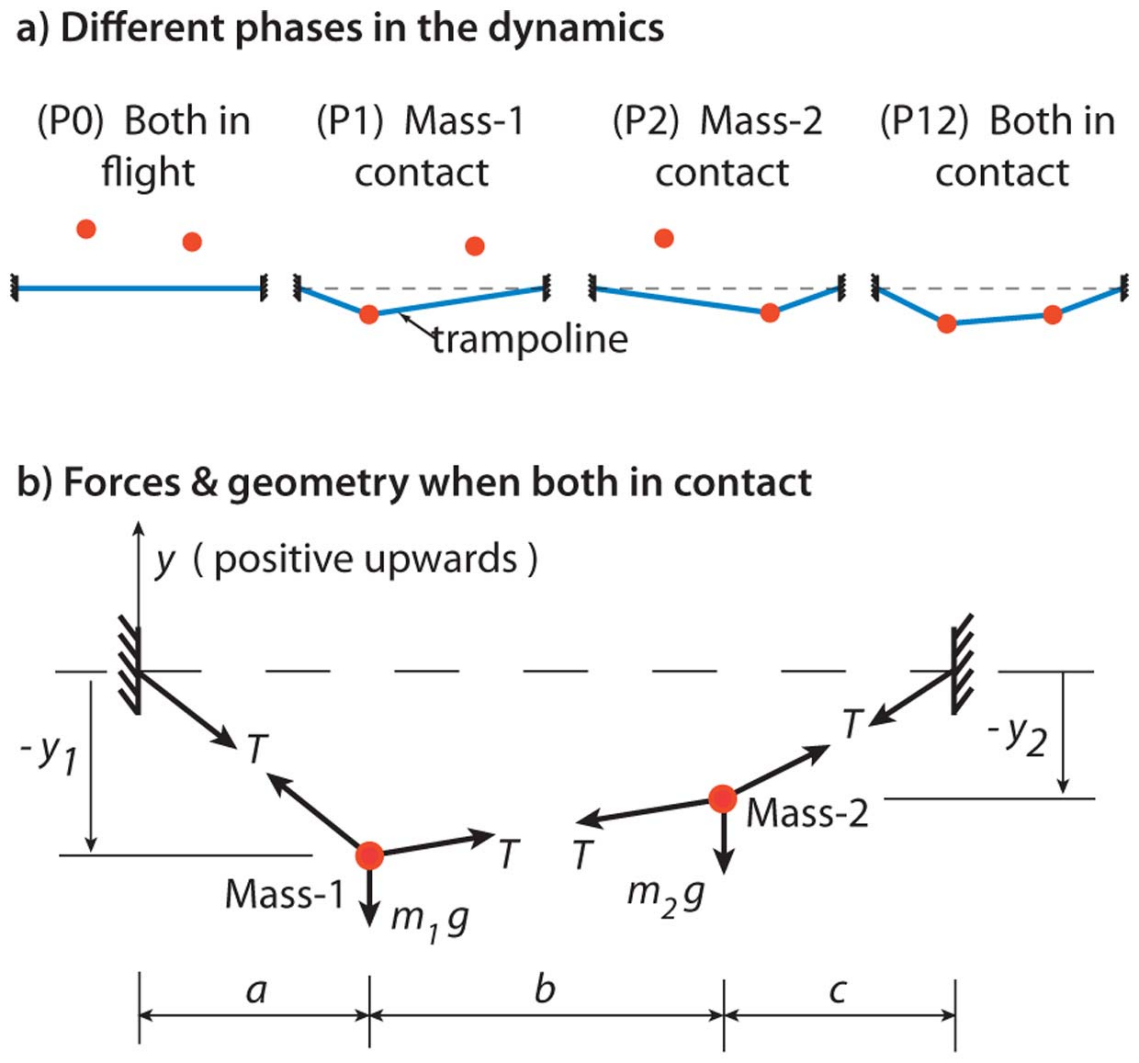
Simple model of two people or two balls bouncing on a trampoline, as two point-masses on a massless trampoline. a) The system can be in one of four phases: neither mass in contact with the trampoline (P0), only mass-1 in contact (P1), only mass-2 in contact (P2), and both masses in contact (P12). b) The geometry of the system is shown, along with the forces on the masses when both are in contact with the trampoline.

We idealize the two players as particles with masses 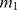 and 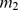 bouncing on a trampoline, modeled as a taut massless string of length 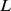, as shown in Figure 3a–b. The two masses are at horizontal distances 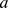 and 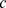 respectively from the nearest fixed ends of the trampoline; the distance between the masses is 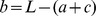. The trampoline is at a large initial tension 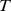, so that the tension in it does not change to first order when deformed (Fig. 3b). The particles do not slip against the trampoline, and we neglect the horizontal forces on the particles, so that the motion of the particles is purely vertical, for all time.

The vertical position of the two masses are denoted 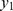 and 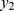, positive upward (Figure 3b). The undeflected trampoline is at 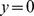. We divide the state space into four phases based on which masses are in contact with the trampoline: P0 (neither in contact with trampoline, both in flight), P1 (only 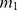 in contact), P2 (only 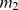 in contact), P12 (both in contact). See Figure 3a. The corresponding equations of motion are:
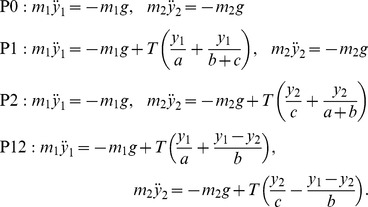
(1)These equations are linear, and assuming small vertical deflections of the trampoline. The total dynamical system, consisting of these four phases patched together, is piecewise linear, and therefore, ultimately, nonlinear and non-smooth. Transition between phases occurs with no discontinuous change in position and velocity. A mass leaves the trampoline when the upward force on it by the trampoline becomes zero while it is moving upward. This take-off event coincides with the trampoline becoming a single straight line to the left and to the right of the mass; that is, the deflection in the trampoline vanishes locally. We assume two take-off or landing events do not happen simultaneously. The trampoline comes immediately to rest when neither mass is in contact; that is, the trampoline has no intrinsic dynamics. These constitutive assumptions are consistent with energy conservation.

The total system energy consists of the kinetic and gravitational potential energies of the two masses, namely 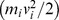 and 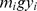 respectively, and the stored energy in the stretched string. The stored energy in the string in the various phases are:
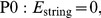
(2)

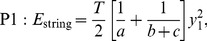
(3)

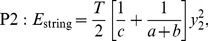
(4)

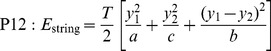
(5)In the following discussions, we will sometimes refer to the “energy of a particular mass,” implicitly partitioning the total system energy between the two masses. The partitioning of the total system mechanical energy into the two masses is clearest when both masses are in flight — then, each mass is associated with the sum of its kinetic and gravitational potential energy. When both masses are in contact with the trampoline, there is no objective partitioning of the total energy between the two masses. When exactly one mass is in contact with the trampoline, we use the convention that the mass that is in contact gets credit for the energy stored in the string.

See Materials and Methods for how this non-smooth dynamical system is simulated.

### Passive dynamics predicts energy transfer and complex dynamics

For the following simulations of the above model pertaining to bouncing people (as opposed to bouncing tennis balls described later), we use the following parameters: 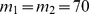 kg, 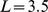 m, 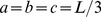, and 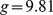 ms^−2^. The vertical stiffness of the trampoline at its midpoint 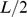 is 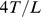. We picked tension 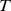 such that this midpoint stiffness was equal to 5000 N/m, roughly the secant stiffness of the trampoline described in [1].

**Figure 4:**
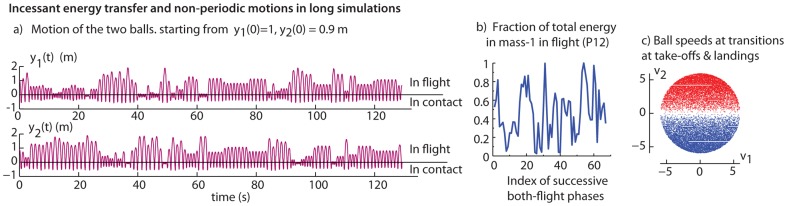
Long-term bouncing dynamics. a) The motion 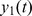 and 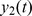 starting at rest from initial conditions 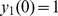 and 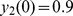. b) The total energy (kinetic+gravitational potential) in mass-1 when both masses are in flight, as a fraction of the total energy. c) The state of the system when the right mass either just takes off (red dots) or when the right mass just lands (blue dots), when the left mass is already in the air.

Before considering a single bounce and the energy transfer in greater detail, we examine simulations of the passive dynamical system for a long time period. Simulating this dynamical system from any generic initial condition (which does not result immediately in the two masses touching the trampoline simultaneously in the first bounce results) in a complex non-periodic bouncing motion of the two masses, as shown in Figure 4a. Also, see video of the animation (movie S3).

The two masses repeatedly exchange energy with each other, sometimes one mass bounces higher and sometimes the other mass bounces higher: Figure 4b shows the fluctuating energy content in mass-1. Thus, over a long enough simulation, if the “game” is stopped at some random moment sufficiently far into the future, there is equal likelihood of either player “winning” i.e., having more energy. The energy transfer between masses occurs when the masses are in simultaneous contact with the trampoline, performing work on each other through the trampoline. Thus, it appears that the passive model is sufficient to explain the energy transfer.

To be clear, while the mechanics of a single bounce interaction of the two masses may be comparable to that of the interaction between humans on a trampoline, the details of the long-time simulation may not be of direct applicability to long-time human bouncing. We discuss this long-time simulation further for its own intrinsic dynamical properties.

In a single long simulation, the state of the system appears to come arbitrarily close to almost every region of the accessible phase space, consistent with energy conservation. Figure 4c gives a scatter-plot of the snap-shots of the state, in a single long simulation lasting about 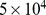 phases, at transitions between phases P0 and P2: when mass-2 lands (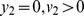, red dots) or takes off (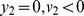, blue dots), with mass-1 already in the air (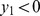). The interior of the disk in Figure 4c is the set of all possible states consistent with constant total energy. We found that the histogram of energies for each mass over time is not a constant function of energy, but has a minimum near symmetric bouncing when each mass has half the energy. Indeed the thin slivers of empty regions in Figure 4c near 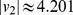 corresponds to states at which each mass has exactly half the energy – in particular, 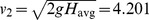 where 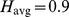 is the average height of the masses at initial condition. (We do not know if the system is ergodic [2].)

The complex dynamics observed for this dynamical system is not entirely unanticipated. A well-studied dynamical system is a mass bouncing, elastically or inelastically, on a much more massive paddle oscillating vertically and exactly sinusoidally [3], [4]; this system is known to be chaotic in certain parameter regimes. Note that this mass on an oscillating paddle system can be obtained as a distinguished limit of our dynamical system by making 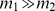 and by ensuring that 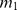 never has a flight phase, so that it oscillates exactly sinusoidally. More recently, apparently unaware of this earlier work, similar chaotic dynamics were observed and analyzed for water droplets bouncing on a fluid surface, acting as a trampoline [5], [6]. One qualitative difference between these earlier systems and the two mass system consider in this article, is that typically the paddle or the fluid trampoline in these systems is oscillated using external energy input, so that the total system energy need not be constant.

### Maximizing energy transfer

In this section (and in the Appendix S1), the passive bouncing model will be used to evaluate the effectiveness of various variables that players could control in order to gain energy from their opponent and win the “seat drop war” game. Players can control their jump timing relative to their opponent, their energy at impact, and the distance between themselves and the opponent. We find that players should aim to contact the trampoline when their opponent has maximally deflected the trampoline (half of the contact time), and should attempt to have more energy than the opponent (either due to larger mass or higher jump). The transfer of energy between players is larger when the players are closer together, than when they are farther apart.

**Figure 5:**
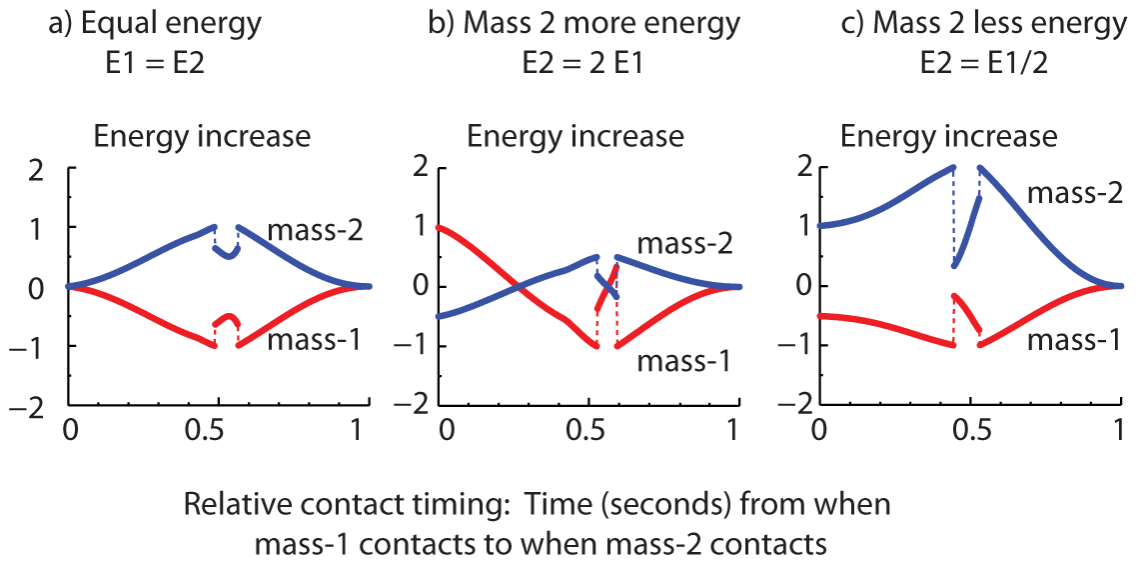
Energy transfer and contact time-lag. Energy increase in mass-1 (red) and mass-2 (blue) as a function of the impact time-lag. The masses are equal and are placed symmetrically on the trampoline, so that 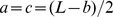. The separation between the masses 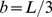. a) The initial energies 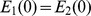 are equivalent to dropping from rest from a height of 1 m. b) Mass-1's initial energy 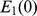 is the same as for panel-a, but 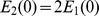. c) Mass-1's initial energy 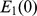 is the same as for panel-a, except mass-2's energy is 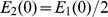.

Energy can get transferred only when both masses are in simultaneous contact with the trampoline. Without loss of generality, consider the situation in which mass-2 lands on the trampoline when mass-1 is already in contact, so that the two masses are in simultaneous contact with the trampoline for a while. Now simulate the system forward in time until both masses are in flight again i.e., phase P0 is reached. We examine the energy increase in the two masses when P0 is reached, as a function of the time difference between when mass-1 makes contact and mass-2 makes contact with the trampoline — the contact time-lag (Figures 5a–c). The energy increase in the two balls is normalized by the pre-contact energy of each mass (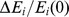), and the contact time-lag has been normalized by the contact period of mass-1 in the absence of interference by mass-2. The model parameters are as noted in the previous sub-section, including 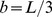, except when specifically overridden below. An alternative version of the plot would record the energies at the first moment one of the balls begins flight. In this alternate version, the ball still in contact will get credit for the stored elastic energy in the string. We note that this version of the plot (not shown) looks slightly different from the plots shown, as the ball in flight has the opportunity to re-contact the trampoline before the other ball takes off, providing further opportunity for energy exchange.

The energy increase in Figure 5a–c are discontinuous functions of the contact time-lag. This discontinuity arises because we record the energy transfer only at the first transition to phase P0 after mass-2 lands. The number of phases that the system goes through before reaching phase P0 can depend on the initial conditions. Thus, on one side of a discontinuity, a mass barely takes off, with close to zero velocity. And on the other side of the discontinuity, this same mass comes very close to taking off, but does not have enough energy to do so, resulting in the system passing through more contact phases phases, P1, P2, and P12, before phase P0 can happen.

In Figure 5a, the masses had initially the same energy. So the normalized energy increases in the two masses are mirror images of each other about the x-axis because of energy conservation (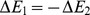), and because they have been normalized by the same quantity (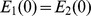).

Here, the mass that lands second, namely mass-2, always gains energy and mass-1 always loses energy, whatever the contact time-lag (Figure 5a). As would be expected, we see in Figure 5a that the energy transfer is close to zero when mass-2 lands when the mass-1 has just landed (close to symmetric simultaneous bounce), or about to take off (close to no interaction). More significantly, we see that the normalized energy increase of mass-2 reaches unity (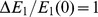), around when the time-lag is about half of mass-1's contact period. That is, if mass-2 makes contact when mass-1 is approximately half-way through its bounce, *the energy transfer is essentially 100% in a single bounce*.

When such complete energy transfer occurs, mass-2 makes contact when mass-1 just starts to rise or just before it starts to rise (note the two contact time-lags on either side of contact timing = 0.5 for which the energy transfer is perfect). As mass-2 pulls the string down, mass-1 remains in contact for a brief while and then leaves contact with an upward velocity, earlier than it would otherwise have in the absence of mass-2. This mass-1's upward velocity 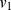 is such that, if left alone, the mass-1 would just about reach y = 0 at roughly the same time that mass-2 leaves contact, so that both masses are in flight with mass-1 with close to zero energy.

**Figure 6:**
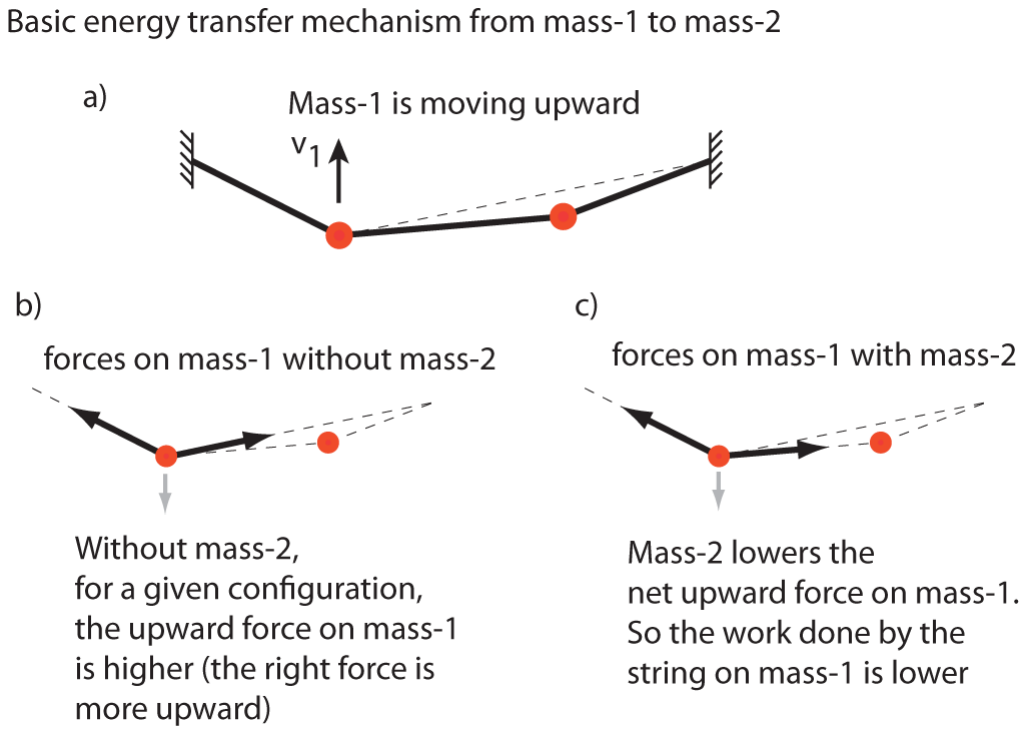
Basic mechanism of passive energy transfer from mass-1 to mass-2. When mass-1 is moving up, the presence of mass-2 lowers the work done by the string on mass-1. Thus mass-1 takes off with lesser upward velocity than if mass-2 had not interfered.

The basic energy transfer mechanism can be most simply understood using an ‘instantaneous argument’ as illustrated in Figure 6. Given a state as in Figure 6a, in which mass-1 is rising while in contact with the string, the presence of mass-2 lowers the upwards vertical force on mass-1 (Fig. 6c) compared to when mass-2 is not in contact (Fig. 6b, keeping position of mass-1 fixed). Thus, for any given upward 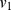, the corresponding acceleration 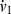 and the instantaneous positive power on mass-1 by the string would be lower than if mass-2 were not in contact. If this situation persists until mass-1 takes off, it would take off with an upward speed smaller than if mass-2 had not interfered. Note that the situation described in this ‘instantaneous’ argument need not persist in general until phase P0 is reached — the bounce dynamics can be quite complex in certain parameter regimes. This heuristic ‘instantaneous’ argument is likely most directly applicable when mass-2 lands on the string when mass-1 is just about to take off, when it is likely that the situation presented in Fig. 6a would persist until at least mass-1 takes off.

The details of the energy transfer's dependence on the contact time-lag can be complex and dependent on various other system parameters including differences in energy of mass-1 and mass-2 before contact (Figure 5b–c). When mass-2 has twice mass-1's initial energy (Figures 5b), mass-1 gains half of mass-2's energy when both contact simultaneously (contact time-lag = 0). In contrast, when mass-2 has half mass-1's initial energy (Figures 5c), mass-2 gains half of mass-1's initial energy when they contact simultaneously (contact time-lag = 0).

Even though the motion is governed by relatively simple linear differential equations in each phase, an analytical treatment to obtain the dependencies in Figure 5 was found to be cumbersome (although likely feasible) because the differential equations had to be integrated until a certain event happened (namely one or both masses taking off) and patched together, rather than simply integrated until a particular time.

In Appendix S1, we discuss energy transfer between the balls under some simplifying limits: (1) Large energy “collisional” limit, in which the collision consequence becomes independent of gravity and many other quantities; (2) limit of masses very close to each other, in which the mass in contact leaves contact when the other mass makes contact; and (3) limit of one mass much higher than the other, in which case the energy transfer scenario is similar to that of a “freshman physics” demonstration involving dropping a small ball sitting atop a big ball, from rest, onto the ground [38], [39].

### Stability of symmetric bouncing

When the masses are equal (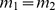) and symmetrically positioned (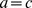), a symmetric periodic motion is achieved by dropping the two balls from the same height. There is a one-parameter family of such symmetric motions parameterized by the initial height. In numerical simulations, we find that a generic small perturbation of the initial conditions for such symmetric bouncing leads to the two balls making contact not quite simultaneously, and this asymmetry grows with time, eventually leading the motion to be far away from symmetric bouncing.

**Figure 7:**
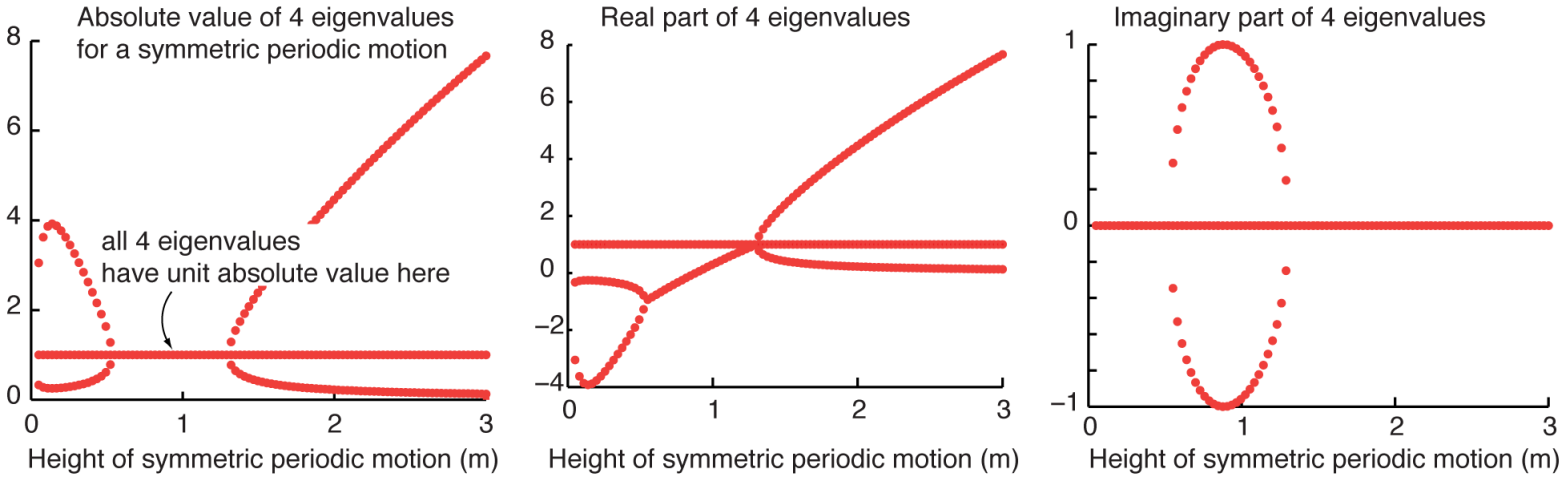
Stability of symmetric passive bouncing. The four eigenvalues of the Jacobian (Floquet multipliers) corresponding to the mapping of the state over one period of the periodic motion: symmetric bouncing for symmetric masses. The product of the eigenvalues was equal to 1 (with an error of about 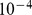). Two eigenvalues are equal to 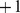. In the intermediate regime shown, all four eigenvalues, two of which are complex conjugates and reciprocals of each other, have unit absolute values. At other regimes, one eigenvalue has magnitude greater than one, implying linear instability.

The stability of symmetric motion is examined by considering the properties of a time-period-based ‘Poincare map’ [3], [7] as described below. (A state-section-based Poincare map has closely related properties.) Say the two balls are dropped from height 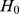 and bounce periodically and synchronously with period 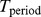. We define our Poincare map as the mapping of states 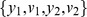 at time 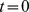 to time 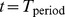. We approximate the Poincare map's Jacobian about the fixed point 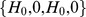 using a central difference scheme. Even though the map is perhaps not arbitrarily differentiable (because the differential equations are non-smooth), the map appears once continuously differentiable, as multiple finite difference approximations using random state perturbations give identical eigenvalues for the Jacobian, up to numerical errors. See Figure 7 for the eigenvalues as functions of bouncing height 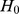. The Jacobian has two unit eigenvalues (equal to +1) for any 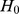 because of energy conservation. We further noticed that for all heights 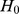, the two non-unit eigenvalues, real or complex, were reciprocals of each other (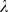 and 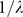), and therefore, the product of all four eigenvalues equals one (true numerically, up to error 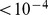). These properties follow from the map being “symplectic” [8], as the map is derived from a Hamiltonian (energy conservative, holonomic) system [25]. For most initial heights 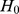, the Jacobian has two non-unit real eigenvalues, with one real eigenvalue greater than one in absolute value, and the other real eigenvalue less than one. Thus symmetric bouncing displays obvious linear instability for these heights. For a small range of heights, the two non-unit eigenvalues were complex conjugates with absolute value equal to one, within numerical error, about 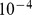, suggesting “spectral stability.” We did not perform any similar analyses that take the non-smooth dynamics into account more carefully. Nevertheless, we note that for all heights, even for heights 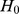 where all eigenvalues seem to have unit magnitudes, long-enough numerical simulations eventually take the system far away from symmetric bouncing. For these simulations, we used a high accuracy integrator that preserved energy at a level of 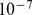 over long durations of integration, but not a ‘symplectic’ integrator [9].

### Game-theoretic analysis: bouncing as a zero sum game

We began this article with a game played on the trampoline as motivation, but all the analysis so far has been of passive mechanical models. Consider, for instance, two players dropping from the same height, with legs extended. Say player 1 knows that player 2 will bounce passively. Then, player 1 can bend her knees during flight, just enough so that she will land slightly after player 2, gaining most of player 2's energy and winning the game. But what if player 2 is thinking strategically as well, trying to time her landing just right so that she can gain all of player 1's energy? What strategy should a player adopt knowing that the other player is also thinking strategically? A rational analysis of such strategic interactions is the purview of *game theory*. In this section, we examine two people jumping on a trampoline from a game theoretic perspective.

For the reader unfamiliar with game theory, we recommend [10] for a non-technical introduction, and [11] for a more advanced mathematical treatment and precise definitions. Game theory is broadly applicable to analysis of any strategic interaction, be it between humans, other animals, computer software – indeed, between any set of agents, such that the consequences of any one agent's action are affected by the actions of other agents. Game theory, pioneered initially by von Neuman [12], has been applied, for instance, to the arms race [13] and to the evolution of species and animal behavior [14].

#### Model with legs

**Figure 8:**
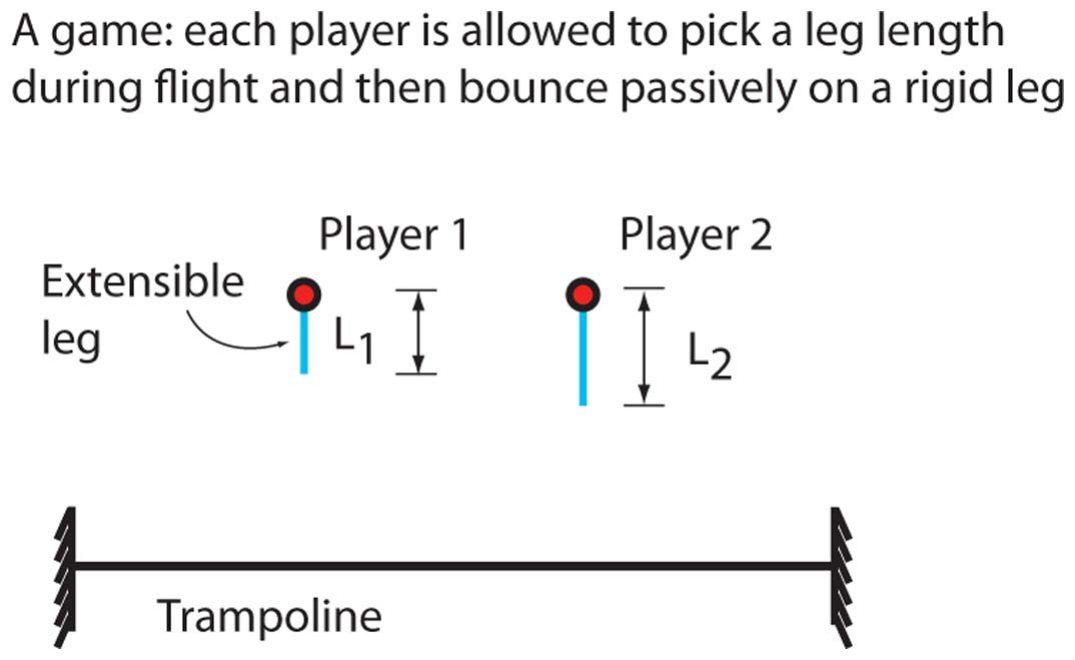
Bouncing as a zero sum game. a) Two players drop from the same height and they can pick a rigid leg length to modify their contact times. b) The normalized energy increase in mass-1 (the payoff function) as a function of the leg length choices of the two players, when the two players drop from an initial height of 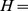
2 m. The vertical line (blue/red) shows the minimax and maximin strategy that coincide. c) The payoff function when initial height is 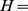
0.9 m. For this height, the pure minimax (denoted player 2) and maximin (denoted player 1) strategy do not coincide. The second panel shows the optimal probabilities corresponding to mixed minimax strategies. For these calculations, we used parameter values pertaining to people, as used earlier (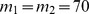
kg, etc).

Consider two players modeled as point-masses as shown in Figure 8, now with mass-less legs that the players can extend and contract within a range of lengths. The leg lengths are respectively 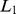 and 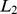, and 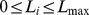. We assume that the leg lengths are picked before contact, and during a bounce, the legs are completely rigid and perform no mechanical work. After interacting with the trampoline, eventually, both players reach flight phase (phase P0) and the leg lengths go back to zero. The object of the game, then, is for each player to pick their leg length 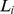 so that their energy is maximum when they reach phase P0, when both are in flight again.

We analyze the energy transfer over only one bounce; that is, over only one complete interaction through the trampoline. Strategies for maximizing energy transfer over multiple bounces were not considered. Picking a non-zero leg length has two possible effects for a player: (1) It makes the player contact the trampoline earlier, thus altering the contact timing relative to the other player. (2) It reduces the initial effective potential energy of the player.

#### A zero-sum game

Player 1 wishes to maximize her energy increase 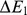 and player 2 wishes to maximize her energy increase 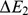. For this game, in the language of game theory, the energy gain after a bounce is the players' *payoff* and the leg length choice during flight is their *strategy* or *move*. This is a *continuous game* [15] in that the players pick continuous-valued variables 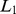 and 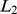. The energy gain functions 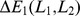 and 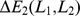 are called *payoff functions* or payoff surfaces.

Because energy is conserved in this system, we have 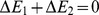. Thus, player 1's energy gain is player 2's energy loss and vice versa. Games such as these, in which the total payoff to all players add up to zero, in which one player's gains exactly equal total losses of the other player(s), are called *zero-sum games* or *strictly competitive games* [11]. Thus, instead of stating that player 2 wishes to maximize 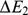, we could equivalently state that player 2 wishes to minimize 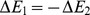.

**Figure 9:**
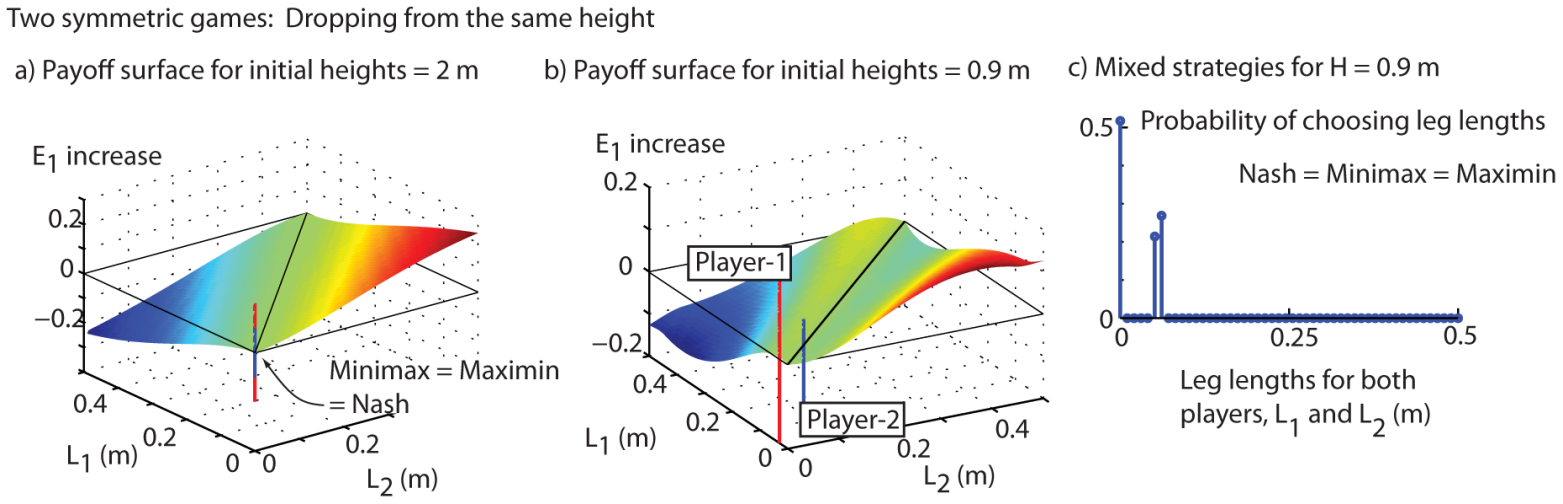
Symmetric games: Dropping from the same height. a) The normalized energy increase in mass-1 (the payoff function) as a function of the leg length choices of the two players, when the two players drop from an initial height of 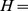
2 m. The vertical line (blue/red) shows the minimax and maximin strategy that coincide. c) The payoff function when initial height is 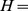
0.9 m. For this height, the pure minimax (denoted player 2) and maximin (denoted player 1) strategy do not coincide. The second panel shows the optimal probabilities corresponding to mixed minimax strategies. For these calculations, we used parameter values pertaining to people, as used earlier (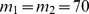
kg, etc).

The payoff functions for our problem (namely, energy transfer 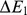) are shown as surfaces in Figure 9a–b, for two different cases in which the two players start at rest from identical initial heights 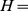
0.9 m and 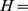
2 m respectively. To construct these payoff surfaces, we discretized the continuous space of strategies, by using a 100×100 grid of 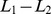 pairs, each ranging from 0 to 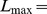
0.5 m. We computed the energy transfers for each 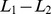 pair on this grid, by performing numerical integrations similar to those that produced Figure 5. This gives a 100×100 payoff matrix, a discrete approximation to payoff function 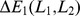.

Given this zero sum game, how should players pick their strategies? Next, we discuss two kinds of game-theoretic solution strategies, namely, deterministic and probabilistic (mixed).

#### Deterministic (pure) strategies

If the player 1 chooses her leg-length 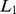 first and player 2 chooses 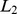 second — with full knowledge of player 1's choice — then player 2 will pick the 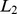 that minimizes the 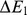 for the already chosen 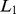. Thus, when player 1 chooses first, she would pick the 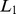 for which the 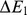 has the maximum minimum. That is, she performs the following optimization problem:
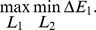
This is the *maximin strategy* for player 1. Similarly if player 2 chooses her leg length first, she will pick the *minimax strategy*, which solves the following optimization problem:
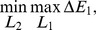
or equivalently,
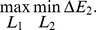
When each player picks a strategy deterministically as above, they are called *pure strategies*.

When the two players have to pick their strategies (leg lengths) simultaneously, another solution concept called the *Nash equilibrium* [16] is more appropriate. An ordered pair of (pure) strategies 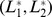 is called a Nash equilibrium if each player cannot improve her value by unilaterally changing her strategy, as the other keeps her strategy fixed. That is, 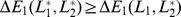 for every 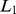 and 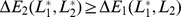 for every 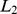. Nash equilibrium captures a idea of stability in the space of possible strategies; once found, neither player has an incentive to choose any other strategy, assuming the players do not cooperate. Note that this notion of stability is different from that discussed earlier in Figure 7 in the context of differential equations. A Nash equilibrium might or not exist in the space of pure strategies.

#### Probabilistic (mixed) strategies

When the two players have to pick their strategies simultaneously, without knowledge of the other player's strategy, it is appropriate to not just consider the deterministic pure strategies as above, but expand the space of strategies to allow probabilistic strategies – called *mixed strategies* [10], [11]. Here, each player picks randomly from the set of possible strategies, with a particular fixed probability distribution. In our case, we assume that player 1 picks her leg length using a fixed probability distribution 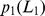 and player 2 picks her leg length with probability distribution 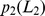.

The mixed Nash equilibrium is the ordered pair of mixed strategies such that a player cannot improve the expected value of her payoff by changing her mixed strategy unilaterally, while the other player keeps her strategy fixed. Given the probability distributions, the expected value of the payoffs 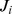 are, respectively,
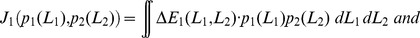
(6)

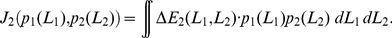
(7)Thus, for a mixed Nash, we seek the two probability distributions 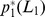 and 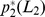 such that
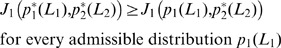
and
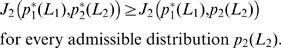
In addition to Nash equilibria, analogous to the minimax problem for pure strategies, one can define a minimax problem over mixed strategies. For instance, player 1 will now solve the following maximin problem:
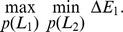
For zero-sum games, the *minimax theorem* due to von Neumann [11] shows, remarkably, that the solutions to maximin and the minimax problems over mixed strategies are equivalent; one obtains the same optimal mixed strategy and the same optimal payoff for each player by solving either the minimax or the maximin problem. Further, for zero sum games, the mixed Nash equilibrium is equivalent to the mixed minimax strategy [11]. Thus, only one of minimax, maximin, or Nash need be computed.

### Strategies and game-theoretic equilibria for bouncing on a trampoline

We compute the game theoretic solutions by first reducing the continuous game to a finite game by discretizing the space of leg length strategies: 50 leg length choices for each player, so that the payoff function is a 50×50 payoff matrix. Then, the computation of the pure minimax/maximin strategies reduce to finding the minimum or maximum of rows and columns (as appropriate) of the payoff matrix. The computation of the mixed minimax or Nash equilibrium can be reduced to a linear programming problem: that is, the minimization of a linear function subject to linear equality and inequality constraints [17]. See *Materials and Methods* for more methodological details. The unknowns in this linear optimization problem are the probabilities at which the various 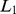 and 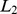 values are chosen by the respective players. A mixed Nash equilibrium always exists for a game with finitely many strategies; and this is true of our game, once we have reduced our continuous game to a finite game.

#### Pure strategies

For our bouncing game, the pure maximin and minimax strategies are shown by vertical blue and red lines, overlaid on the payoff functions in Figure 9a and 9b. The minimax and maximin (the vertical lines) happen to coincide at 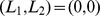 for falling from a larger height (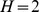 m, Figure 9a). That is, starting from this height, it is best to not extend the legs. On the other hand, the minimax and the maximix strategies do not coincide for a smaller drop height (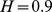 m, Figure 9b): the maximin strategy for player 1 has a small non-zero 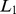, with 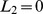; the minimax strategy for player 2 is the mirror image of the maximin strategy (reflected about 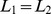). Other than to draw attention to the differences in the shape of the payoff functions in the two cases, namely Figures 9a–b, we cannot provide a simple mechanically intuitive explanation for this qualitative difference in pure strategies depending on initial energy.

#### Mixed strategies in symmetric bouncing games: No expected gain for either player

When the initial heights are equal and all other parameters are symmetric, we have a ‘symmetric’ zero-sum game [11], defined by 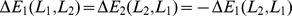, or in terms of the corresponding payoff matrices, we have, 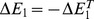. Symmetry is relevant because it implies some properties of the game's solution. When all parameters are symmetric, including equality of the initial heights, perhaps not surprisingly, neither player can have a strategy (pure or mixed) that guarantees a non-zero expected energy gain. That is, the expected value 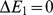, and both players will, on average bounce back up to the same height. Indeed, a standard theorem for symmetric zero sum games states that the expected value for minimax mixed strategies is zero [11].

When the initial heights both equal 2 m, the optimal mixed strategy coincides with the pure strategies, namely 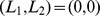. For a lower initial height (both equal 0.9 m), when the pure strategies did not coincide (Figure 9b), the optimal mixed strategy for the two players, namely, the probability distribution over the allowed leg lengths, is shown in Figure 9c. We see that the players will have to choose zero leg length with a probability of about 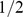 and a leg length of about 0.6 with a probability of about 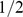 — more precisely, two neighboring grid points with leg length around 0.6 have about 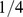 each. That the probability is distributed over two neighboring grid points is likely a discretization artifact, with a ‘correct leg length’ to be used being between the two grid points.

#### Mixed strategies in asymmetric bouncing games: Rich get richer

When a zerosum game is not symmetric, there is no longer a theorem that states that the expected value of the payoffs will be zero. The bouncing game is not symmetric (generically) if the players start with different initial heights from rest, different initial velocities, have different masses, or have asymmetric positions along the trampoline (lengths 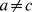). We considered all these asymmetries, one at a time, and computed the expected payoff of each player's minimax strategy, also the Nash equilibrium.

**Figure 10:**
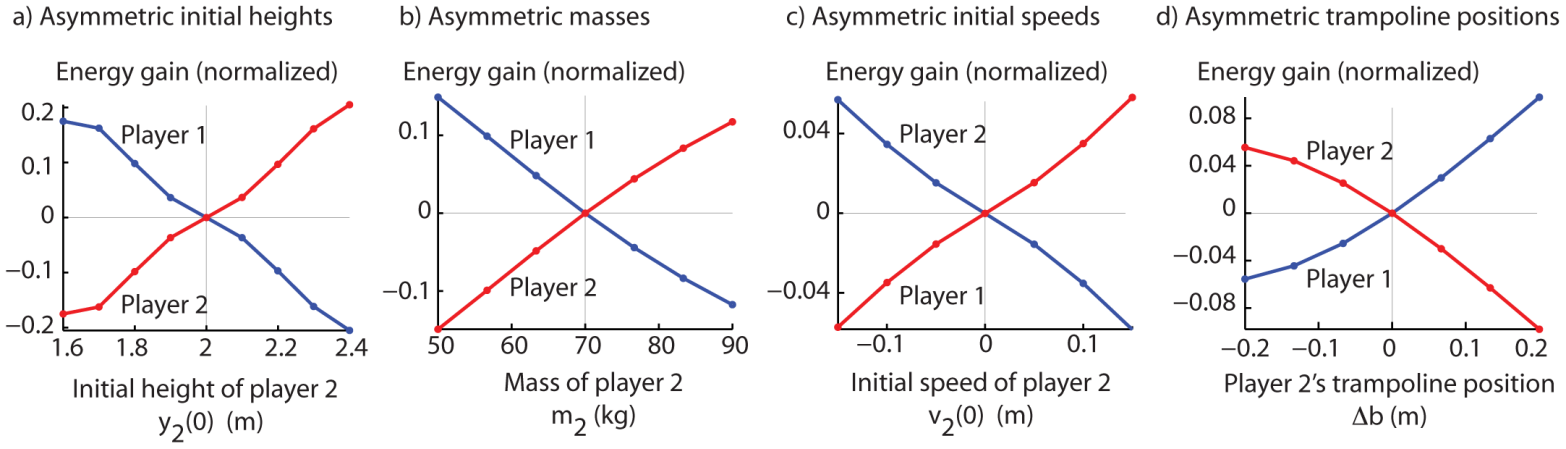
Asymmetric games. All parameters are kept at their default symmetric values, except for the one parameter that is varied for each panel. a) Players drop from different initial heights. Player 1 has initial height 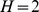 m; player 2's initial height is varied as shown. All else is symmetric. The player that starts higher, that is, has higher initial energy, gains even more energy in a single bounce, further increasing her energy. b) Players have different masses. Player 1's mass 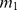 is kept at 70 kg and player 2's mass 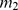 is varied as shown. Again, the player with greater initial energy gains even more energy. c) Players have different initial speeds. Player 1 starts at rest and player 2's initial speed 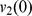 is changed. When player 2 has a positive or upward initial speed, it lands second and gains energy from player 1. d) Players have asymmetric positions on the trampoline: player 2's position is moved to the right by 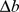. We find that the player that is closest to its end of the trampoline loses more energy.

For Figure 10a, we considered the two players dropping from rest from slightly different initial heights, holding all other parameters symmetric as before; we have 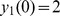 m, and 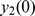 is varied between 1.6 and 2.4 m. For each of these initial heights, we first compute the payoff surface and then obtain the mixed Nash/minimax solution by linear programming. For this range of initial heights, we find that the mixed Nash/minimax solution always had the following property: the player that initially has a higher energy – that is, drops from a higher initial height – had an expected energy gain. That is, the rich get richer. Thus, any small initial energy asymmetry only grows in time, making synchronous bouncing unstable (unstable in the sense of Figure 7 which pertains to time-evolution of the bouncing – rather than unstable in the sense of Nash). Similarly, in Figure 10b, we consider the effect of mass 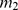 ranging from 50 to 90 kg, while keeping everything else symmetric. Again, we find that the mixed minimax/Nash is such that the player that has the greater mass – and therefore starts with more energy – stands to gain even more energy in a single bounce.

For Figure 10c, we keep everything symmetric except change the initial speed 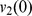 of player 2, keeping 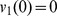. Thus, the initial energy of player 2 is greater than that of player 1 for all the cases considered here. Nevertheless, one player can gain energy from the other — we see that the player with the greater downward speed – that is, the player that lands first – gains more energy. Note that this trend is consistent with that observed earlier with the passive bouncing – for instance, similar to Figure 5a, which however has the two masses start with equal energy.

Finally, for Figure 10d, we keep everything symmetric except for the horizontal position of player 2 along the trampoline. That is, we keep the position 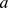 of player 1 fixed, change length 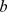 by 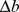 and change length 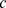 by 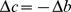. Here, the players start with the same energy, and we find that the player closer to the center gains more energy.

Overall, the results for asymmetric games in Figure 10 are similar to those obtained earlier for passive bouncing. This is because even though the panels in Figure 10 were obtained by computing the mixed minimax/Nash strategies, ultimately, the ‘mixed’ strategies obtained were pure. That is, almost all computed strategies for Figure 10 involved using 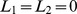 with unit probability. The only exception we found was for especially large and small 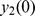 in Figure 10a, for which the computed mixed strategies actually had a non-trivial probability distribution spread over the different available leg lengths.

### Many balls bouncing

**Figure 11:**
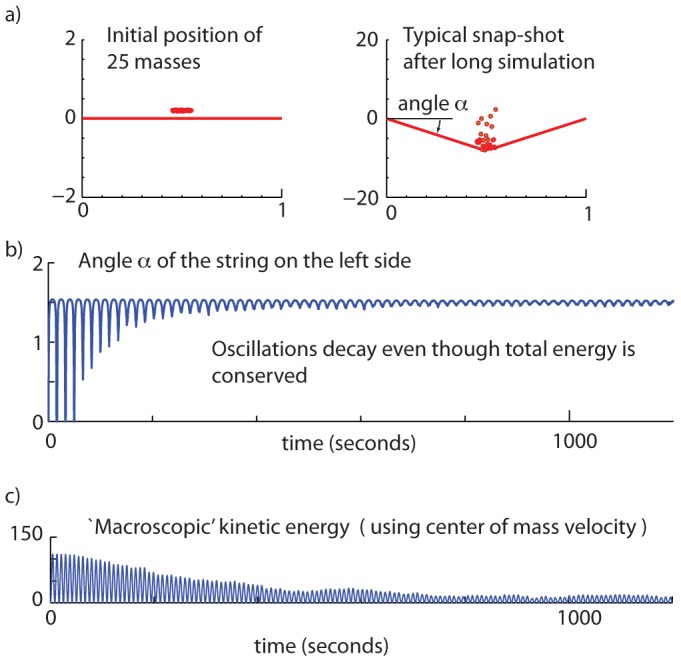
Statistical mechanics of many balls bouncing passively. a) Twenty five balls are dropped from approximately but not exactly the same height. b) The angle of the string is shown as a function of time. Note that the string oscillates macroscopically initially, as the masses bounce together coherently on it. However, eventually this macroscopic coherent motion of the masses and the coherent oscillatory string motion gets ‘damped out’ with the energy getting transferred to incoherent motion of the masses. c) A ‘macroscopic’ kinetic energy computed as 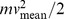 with mean ball velocity 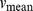 decreases with time, as the masses' velocities cancel each other more.

As an aside for future work, we generalized our two-ball simulation to the the bouncing of 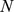 balls on the trampoline (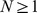). While we did not examine this system in great detail, we found that even with small numbers of balls, say about 10, the system starts to exhibit properties reminiscent of macroscopic statistical mechanical systems. For instance, Hamiltonian systems with very large number of degrees of freedom, even though energy-conservative, are capable of phenomena analogous to ‘damping’ — conversion of macroscopically observable kinetic energy to internal degrees of freedom. Figure 11 demonstrates this phenomenon in a 25 ball system. Here, initially the macroscopically coherent motion of the masses, moving together as a whole, gets converted into largely incoherent motion of the individual particles. Thus, we see that the oscillation amplitude of the string decays, even through the total energy in the masses is conserved. The mean velocity of the masses (center of mass speed) decreases, while the kinetic energy relative to the system's center of mass increases. See section on Materials and Methods for simulation details. See video animation in movie S4.

## Discussion

In this article, we have examined the mechanics of energy transfer between two masses bouncing on a trampoline and various aspects of their corresponding dynamics. First, we demonstrated this energy transfer with a table-top set-up, consisting of two balls dropped onto a mini-trampoline landing almost simultaneously. We find that sometimes, when the timing between the two balls landing is just right, one ball bounces much higher than the other. Next, we devised a simple mathematical model of two masses bouncing passively on a massless trampoline with no dissipation. With this mathematical model, we showed that with specific landing conditions, it is possible to transfer all the kinetic energy of one mass to the other through the trampoline, explaining the dramatic energy transfer observed in actual bouncing with humans as well as our table-top demonstration. To our knowledge, we do not know of a prior mathematically based explanation or documentation of this energy transfer. For human-like parameters, starting with equal energy, the energy transfer is maximal when one person lands approximately when the other is at the bottom of her bounce. The passive energy conservative model also has the interesting property that over a long time, the energy shuttles back and forth between the two masses, sometimes almost entirely in one mass and sometimes in the other.

Thus, while the passive model tells us what a player should do (land a little later) to steal the other person's energy in an otherwise passive bounce, it does not tell us what each player would or should do when she knows that the other player is also thinking strategically about the game. We address this strategic interaction question through a game theoretic analysis. To the passive model, we added legs that can change length in flight so as to affect when the player lands and the energy at landing. Starting from both players in flight, we computed the energetic payoff of each player choosing a range of leg lengths before landing. We then computed Nash equilibria and minimax-maximin strategies for the players, which tell us what each player should do given the other player is also thinking strategically. Not surprisingly, we found that if the two players start from the same height, the Nash equilibrium strategies involve neither player gaining or losing energy after a single bounce. However, under asymmetric conditions, one player can gain another player's energy by appropriate choice of leg lengths. For instance, when the two players started from slightly different initial heights, the player that had the greater initial height could pick a leg length that will increase her energy further after a single bounce, for whatever other player does.

Thus, we find that symmetric bouncing is unstable, both for the passive mathematical model and for the game-theoretic version in which leg lengths are chosen strategically by the subjects. The game theoretic model is also able to predict – how body parameters and trampoline location might or not give one or another player a strategic advantage, given the state of the game.

In our game theoretic analysis, we assumed a simplification of the game that preserved total energy conservation, making the game zero-sum. Allowing for active leg work or passive dissipation during contact with the trampoline will make the game non-zero sum. Also, allowing for positive or negative leg work will require appropriately discretizing the space of leg actuation strategies, perhaps similar to that used in other optimization studies of human movement [18]–[20]. Such leg work will let a player adjust the landing timing by adjusting the jump height as well – in addition to leg length change during flight as we have considered here. We considered the strategic interaction over only a single bounce; it may be interesting to examine what the long-term outcomes will be if the players pick their leg lengths before each bounce.

There have been a few specific applications of game theory to physical games played by humans, such as soccer [21], tennis [22], and indeed pursuit games [23]. We have provided a further example in the current paper — an example with rich dynamics, arising out of the hybrid mechanical system consisting of the interaction between the subjects and the trampoline.

Further, while we considered a simple adversarial situation in which each player is trying to steal energy from the other, we could also consider other scenarios that involve a cooperative flavor, in which the subjects are trying to maximize their combined energy or decide how they should cooperate to avoid a non-optimal solution to each, analogous to the *bargaining problem* [16], [24]. This will be relevant mainly in the non-zero-sum setting, perhaps when there is the possibility of each player adding or removing energy from the system by performing leg work.

We found that our mathematical model of the passive bouncing dynamics has complex dynamics, which might be of interest to dynamicists. This system is energy-conservative and Hamiltonian [25], even though only piecewise smooth. Other non-Hamiltonian energy conservative systems, such as an ideal bicycle [20], [21], which is non-holonomic, and a spring-mass model of human and animal running [14], which is non-smooth (and involves some non-passive but energy-neutral external control), have partial asymptotic stability that Hamiltonian systems cannot have (because Hamiltonian systems satisfy Liouvilles theorem of phase-space volume preservation [25].). Perhaps there are variants of our trampoline bouncing model which do have partial asymptotic stability. We conjecture that it may be possible to achieve partial asymptotic stability of symmetric bouncing, by actively changing leg lengths by the two players during flight, but without ever changing the total energy of the system. This would be an example of “cooperative control” [26], albeit in an unconventional energy conservative setting. Indeed, it would be interesting to see if two human players can intentionally stabilize symmetric bouncing. There is an Olympic sport called synchronized trampoline, in which two gymnasts perform the same routine but on neighboring trampolines. Presumably, attempting this sport on the same trampoline would cause the gymnasts' performance degrade substantially, because of the sensitive dependence of the bounce on the contact timings. But perhaps this would be a way to detect asynchrony that may be harder to perceive with the naked eye.

The phenomenon of dramatic energy transfer discussed here has relevance to the mechanics of collisions. The energy transfer here persists even when the trampoline's stiffness is very large and the contact duration very short – our table-top demonstration is already near this ‘collisional’ limit. In this limit, the dramatic energy transfer between the two masses through the trampoline is reminiscent of certain results in the literature on simultaneous ‘rigid-body collisions.’ In particular, simultaneous collisions of nominally rigid-bodies – collisions in which there are multiple points that are making contact at the same time – are known to be often ill-posed. That is, when these simultaneous collisions are ‘regularized’ either by making the contacts happen in sequence or by making the collisions last a non-infinitesimal period with varying time-overlaps between the various contacts, it is found that the details assumed (either the collision sequence or the overlap details) substantially affect the collision consequence [27]–[31]. The current article provides another example of such sensitivities.

Finally, we comment on possible implications to the bouncing games that children and adults play on trampolines. The dramatic energy transfer between players bouncing on a trampoline may have an effect of trampoline injuries. Some epidemiological studies [32]–[34] suggest that a substantial fraction of trampoline injuries are when multiple people were on the trampoline simultaneously. Indeed, official USTA safety manual [35] recommends that more than one person never bounce on the same trampoline. It is possible that the greater injury likelihood with many players on the trampoline could be due to three inter-related effects. First, of course, the large energy transfers between people can produce high bounces that may be harder to control, resulting in injuries. Second, the larger energy transfers are also associated with larger forces on the body, often twice as much as when only one person is bouncing. Third, as is clear from the Figures 5 and Appendix S1 (Figures S1, S2, S3), the amount of energy transfer can depend very sensitively on the contact time-lag, lowering the player's ability to brace for and control the bounce. Thus, while the energy transfers may make multiple people bouncing on a trampoline more fun, they also make such bouncing more dangerous and unpredictable.

## Materials and Methods

### Non-smooth dynamics simulations

The equations of motion (Eq. 1) describing two balls bouncing were integrated in MATLAB using standard ordinary differential equation solvers (ode45 and ode15s with high accuracy specifications 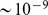). Switching from one phase to another phase of the hybrid system is achieved with the in-built ‘event detection’ capabilities of the ODE solvers, so that the solution is formed as a patch-work of solutions to smooth differential equations. See Programs S1 for the complete MATLAB code.

For 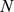 ball simulations, 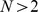, we now present the equations of motion in an algorithmic form. Let 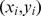 denote the horizontal and vertical positions of the 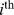 ball, 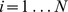. If ball-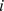 is not in contact with the string, its motion is governed by 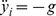. At any moment, the set of balls that are in contact with the string are determined as those with 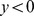. Say there are 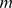 balls in contact and the set 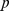 contains the indices of the balls in contact. For instance, if balls 3, 5, and 8 are in contact, 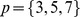 and 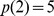.

The equation for 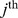 ball in contact (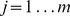) is:
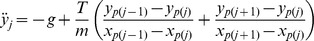
in which, in addition to the above definitions, we have 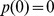, 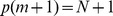. Also, 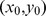 and 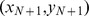 are the fixed positions of the left and right ends of the string. See Programs S1 for the complete MATLAB code. For this ODE solution, we simply integrate over the non-smoothness in the right-hand side of the differential equation, using the stiff solver ode15s. While this is not ideal for solution accuracy (as the solvers assume higher-order differentiability), the adaptive step-sizing keeps the error small enough that energy is conserved over reasonable time-scales. This accuracy seems sufficient for obtaining overall qualitative or statistical properties of the dynamics (as verified by comparing with the more accurate two-ball simulation).

### Table-top demonstration

For the physical table-top demonstration, we used lacrosse and tennis balls dropped onto Gold's Gym mini trampoline, 36 inches in diameter. We did not tune any of the stiffnesses, and our calculations suggest that the effects will be seen on trampolines of vastly different mechanical properties.

### Computing game-theoretic solutions: Pure strategies

As noted, first, we evaluate the payoff function 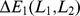 at finitely many 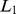 and 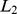 to obtain a payoff matrix. Evaluating the payoff requires solving the differential equations until both players are in flight again. We used 50-element lists of 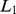 and 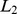, namely 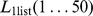 and 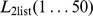, with the lengths ranging from 0 to 0.5 m, to get 50×50 payoff matrices. Let us call the payoff matrix 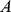 for player 1 and 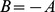 for player 2, to distinguish them from the corresponding continuous functions 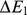 and 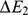. We use the convention that the matrix element 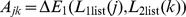, where 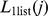 represents the 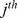 element of the list of 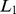
's.

Given the payoff matrix A, we find the maximin strategy for player 1 by first finding the minimum of each row of 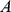; and then finding the row with the maximum minimum. The 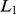 and 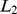 corresponding to those rows and columns give the maximin solution. Equivalently, the minimax strategy for player 2 is found by repeating this same maximin procedure on 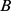. Alternatively, the minimax strategy for player 2 is found by first finding the maximum for every column of 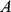 and then finding the column with the minimum maximum.

### Computing game-theoretic solutions: Mixed strategies

The mixed equilibria, as noted earlier, are solved using linear programming, briefly described as follows. Consider the perspective of player 1, who wishes to maximize 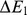. Again, we discretize the continuous space of strategies, so that we can examine finitely many strategies 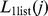 and 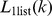 chosen by the two players (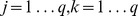, say). The corresponding payoff matrix is 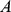, with elements 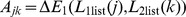. Player 1 chooses strategy 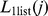 with probability 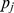 (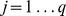). These probabilities 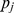 are our unknowns and are found by solving the following maximin problem:
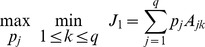
(8)

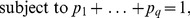
(9)

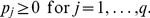
(10)As outlined in [17], [36], in his maximin problem can be converted into a simple optimization problem, with linear objective function and linear constraints – a linear programming problem – by introducing an additional variable 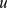 as follows:
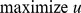
such that
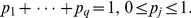
(11)

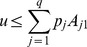
(12)

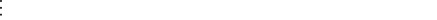
(13)

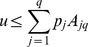
(14)where the solution for variable 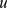 equation gives the expected payoff for player 1. This linear programming problem has 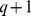 unknowns, namely, the probabilities 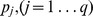 and u. The optimization problem can be solved reliably with provable guarantees using standard software (linprog in MATLAB). See [37] for an introduction to linear programming.

To obtain the probabilities with which player 2 should choose leg length 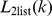, we repeat the above procedure after replacing every occurrence of 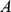 replaced by 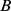. We verified that the expected payoffs for the two players, obtained by solving the problem independently from each player's perspective, are identical up to numerical tolerances.

The MATLAB code used to solve these problems are part of Programs S1.

## Supporting Information

Movie S1 **Video of the people bouncing on a trampoline, playing the game “seat drop war”.** Video features the Dublin University Trampoline Club (bounce@tcd.ie). See also [40].
(MP4)

Movie S2 **Video of table-top experiment, in slow motion.** Two balls are dropped nearly simultaneously onto a table-top trampoline. They rise to same or different heights depending on the time-lag between their contacting the trampoline.
(ZIP)

Movie S3 **Animation of two balls bouncing on an ideal trampoline, obtained by simulating the passive-bouncing energy-conservative mathematical model for a long time.** We see that the two masses exchange energy back and forth over the many bounces.
(ZIP)

Movie S4 **Animation of many balls bouncing on an ideal trampoline, obtained by simulating the many-ball version of the passive-bouncing mathematical model.** Different initial conditions and ball positions are shown.
(ZIP)

Appendix S1 **This appendix contains text and three figures discussing energy transfer under three different simplifying limits (1) when the initial energy of the two masses is large; (2) when the two masses are closely spaced; and (3) when one mass is much heavier than the other.**
(PDF)

Programs S1 **This is a compressed folder, containing MATLAB programs that enable the major simulations and calculations in this article.** In particular, we provide (1) programs which simulate two balls bouncing on an ideal trampoline; (2) programs which simulate many balls bouncing on an ideal trampoline; (3) programs which perform the game theoretic calculations.
(ZIP)

## References

[1] Czaplicki A, Blajer W (2003) Contact modeling and identification of planar somersaults on the trampoline. Multibody System Dynamics 10: 289–312.

[2] Berry MV (1981) Regularity and chaos in classical mechanics, illustrated by three deformations of a circular ‘billiard’. European Journal of Physics 2.

[3] Guckenheimer J, Holmes P (1983) Nonlinear Oscillations, Dynamical Systems, and Bifurcations of Vector Fields. Springer-Verlag.

[4] Tufillaro N, Abbott T, Reilly J (1992) An experimental approach to nonlinear dynamics and chaos. Addison-Wesley.

[5] Gilet T, Bush JWM (2009) Chaotic bouncing of a droplet on a soap film. Phys Rev Lett 102: 014501. 19257198 10.1103/PhysRevLett.102.014501

[6] Gilet T, Bush JWM (2009) The fluid trampoline: droplets bouncing on a soap film. J Fluid Mech 625: 167–203.

[7] Garcia M, Chatterjee A, Ruina A, Coleman M (1998) The simplest walking model: Stability, complexity, and scaling. J BIOMECH ENG TRANS ASME 120: 281–288. 10.1115/1.2798313 10412391

[8] Meiss JD (1992) Symplectic maps, variational principles, and transport. Reviews of Modern Physics 64: 795–848.

[9] Hairer E, Lubich C, Wanner G (2006) Geometric Numerical Integration: Structure-Preserving Algorithms for Ordinary Differential Equations. Springer.

[10] Davis MD (1983) Game Theory: A Nontechnical Introduction. Courier Dover Publications

[11] Osborne MJ, Rubinstein A (1994) A course in game theory. The MIT Press.

[12] Von Neumann J, Morgenstern O (1947) The theory of games and economic behavior. Princeton university press.

[13] Poundstone W, Metropolis N (1992) Prisoner's dilemma: John von Neumann, game theory, and the puzzle of the bomb. Physics Today 45: 73.

[14] Smith JM (1993) Evolution and the Theory of Games. Springer.

[15] Bryson A, Ho Y (1975) Applied Optimal Control. John Wiley, NY.

[16] Nash JF Jr (1950) The bargaining problem. Econometrica: Journal of the Econometric Society 155–162.

[17] Raghavan T (1994) Zero-sum two-person games. Handbook of game theory 2: 735–768.

[18] Srinivasan M, Ruina A (2006) Computer optimization of a minimal biped model discovers walking and running. Nature 439: 72–75. 16155564 10.1038/nature04113

[19] Sheets AL, Hubbard M (2009) Influence of optimization constraints in uneven parallel bar dismount swing simulations. Journal of biomechanics 42: 1685–1691. 19457485 10.1016/j.jbiomech.2009.04.014

[20] Srinivasan M (2011) Fifteen observations on the structure of energy minimizing gaits in many simple biped models. Journal of the Royal Society Interface 8: 74–98. 10.1098/rsif.2009.0544 PMC3024815 20542957

[21] Chiappori PA, Levitt S, Groseclose T (2002) Testing mixed-strategy equilibria when players are heterogeneous: the case of penalty kicks in soccer. American Economic Review 1138–1151.

[22] Walker M, Wooders J (2001) Minimax play at wimbledon. The American Economic Review 91: 1521–1538.

[23] Isaacs R (1999) Differential games: a mathematical theory with applications to warfare and pursuit, control and optimization. Dover Publications

[24] Wong KKL (2010) A geometrical perspective for the bargaining problem. Plos one 5: e10331. 20436675 10.1371/journal.pone.0010331 PMC2859940

[25] Goldstein H, Poole CP, Safko J (1980) Classical mechanics. Addison-Wesley.

[26] Fax J, Murray R (2004) Information flow and cooperative control of vehicle formations. Automatic Control, IEEE Transactions on 49: 1465–1476.

[27] Ivanov A (1995) On multiple impact. J Appl Math Mech 59: 887–902.

[28] Ruina A, Bertram JEA, Srinivasan M (2005) A collisional model of the energetic cost of support work qualitatively explains leg-sequencing in walking and galloping, pseudo-elastic leg behavior in running and the walk-to-run transition. J Theor Biol 14: 170–192. 10.1016/j.jtbi.2005.04.004 15961114

[29] Srinivasan M, Ruina A (2008) Rocking and rolling: A can that appears to rock might actually roll. Physical Review E 78: 066609. 10.1103/PhysRevE.78.066609 19256970

[30] Chatterjee A (1997) Rigid Body Collisions: Some General Considerations, New Collision Laws, and Some Experimental Data. Ph.D. thesis, Cornell University.

[31] Chatterjee A (1999) On the realism of complementarity conditions in rigid body collisions. Non linear Dynamics 20: 159–168.

[32] Black GB, Amadeo R (2003) Orthopedic injuries associated with backyard trampoline use in children. Can J Surg 46: 199–201. 12812242 PMC3211739

[33] Woodward GA, Furnival R, Schunk JE (1992) Trampolines revisited: A review of 114 pediatric recreational trampoline injuries. Pediatrics 89: 849–854. 1579393

[34] Esposito PW, Esposito LM (2009) The reemergence of the trampoline as a recreational activity and competitive sport. Current Sports Medicine Reports 8: 273–277. 19741356 10.1249/JSR.0b013e3181b8f60a

[35] USTA (2004) United States Tumbling & Trampoline Association: Safety Manual, Fourth Edition. Available: http://www.usta1.org. Accessed 2013 Sept.

[36] Ferguson TS (2013) Game theory. UCLA. Available: http://www.math.ucla.edu/~tom/Game_Theory/Contents.html. Accessed 2012 Sept.

[37] Chvatal V (1983) Linear programming. Macmillan

[38] Pattar U, Joshi AW (2002) Head-on collision of two balls revisited. Resonance 7: 67–77.

[39] Cross R (2007) Vertical bounce of two vertically aligned balls. American Journal of Physics 75: 1009–1016.

[40] Dublin University Trampoline Club (2011). Seat Drop Wars (Dublin Area Championships). http://www.youtube.com/watch?v=EyEc-s20qZ8 Accessed 2013 Sept.

