# Supplementary material for: People Bouncing on Trampolines: Dramatic Energy Transfer, a Table-Top Demonstration, Complex Dynamics and a Zero Sum Game
Source: PLoS One. 2013 Nov 13;8(11):e78645. doi: 10.1371/journal.pone.0078645 (PMC3827250; doi:10.1371/journal.pone.0078645)
Supplement: Appendix S1 — This appendix contains text and three figures discussing energy transfer under three different simplifying limits (1) when the initial energy of the two masses is large; (2) when the two masses are closely spaced; and (3) when one mass is much heavier than the other. (PDF) [file pone.0078645.s005.pdf]

Appendix S1

Energy transfer: some limiting cases

In the main article, we have characterized to some extent (Figures 5a-c), the dependence of the post-bounce state on the details of the pre-bounce state.

Large energy "collisional" limit. When the initial (pre-contact) kinetic energy of the two masses is large, many of the parameter dependences vanish, resulting in some simplification. In this limit, which is the same as the tension in the string being very high, we note that there is: (1) no dependence on gravity (equivalent to setting $g \to 0^+$), as the gravity would be much smaller than the elastic forces during contact; (2) no dependence on the masses, only their ratios. (3) no dependence on the stiffness, only the length ratios a/L and b/L; (4) no dependence on the initial energies (or initial heights), only their ratios.

Say the masses are equal, symmetrically placed on the trampoline, and start with the same large energy. Then, the relative energy transfers depend only on the length ratio b/L (Figures 1a-c). Our claim of independence with respect to various parameters in this large energy limit is buttressed by the fact that each of these figures is a superposition of up to 9 different curves, and for many parameter values, varying by more than an order of magnitude or two.

Complete energy transfer seen in Figures 5a-c is not found in this limit (Figures 1a-c); we do not have a simple explanation. When the two masses are far apart, placed near the extremes of the string, their interaction through the string reduces and the maximum energy transfer goes to zero as $b \to L$ (Figure 1c).

Two masses very close together. When the two masses are very close to each other, if mass-2 makes contact when mass-1 is in contact, mass-1 loses contact almost immediately. This is because, mass-2 pulls the string down — and because of its closeness to mass-1, it has to travel a very small distance downwards before the string around mass-1 becomes straight, allowing mass-1 to take off. Often, before both masses are simultaneously in flight (that is, phase P0 is reached), the two masses go through many P1 and P2 phases, interchanging contact, especially when the contact time-lag was very small. The energy transfer until phase P0 is reached, at the high energy limit, is shown in Figure 1b; the oscillations in the energy transfer obtained reflects the multiple phases gone through until P0.

Instead of plotting the energy transfer when phase P0 is reached, it is more illuminating in this case to plot the energy transfer at the moment when one of the balls has taken off (Figure 2a). If mass-1 leaves contact immediately when mass-2 lands, then the energy transferred to mass-2 is whatever was left in the string when mass-2 contacted. This expected equality of the energy transferred to mass-2 and the elastic energy in the string when mass-2 contacted is shown in Figure 2b.

Very different masses. A classic physics demonstration involves placing a tennis or ping pong ball on top of a basketball, and dropping them simultaneously from rest. The dramatic result is that the smaller ball bounces to many times its initial height, sometimes hitting the room's ceiling. This behavior is understood by noting that the basketball's velocity is reversed first, and that the tennis ball is colliding with a basketball that is already moving upward. For elastic collisions, and in the limit of zero mass ratio, the smaller ball rises to nine times its initial height. See [1, 2] for a careful derivation.

Analogously, say $m_2 \ll m_1$, mass-2 makes contact when mass-1 is already in contact, and that the two masses have been dropped from rest from roughly the same initial heights, so the initial energies are very different. For $b = c = L/3$, assuming that mass-2's bounce is short and does not affect mass-1's velocity, we can show that: (1) mass-2 gains most energy if it contacts mass-1 has maximum upward speed, about to take-off; mass-2's energy increase is three times its initial energy. (2) mass-2 loses essentially all its energy if it lands just after mass-1 has landed, when it has the greatest downward speed. These two limits are reflected in Figure 3a, when the contact time-lag is, respectively, close to one or zero.

When the initial energies are equal, $m_2 \ll m_1$, mass-2's bounce now has an effect on mass-1's dynamics, so the above simple intuition falls apart. Energy transfer in this case and in the case when the mass landing second $m_2 \gg m_1$ are shown in Figure 3b and 3c respectively.

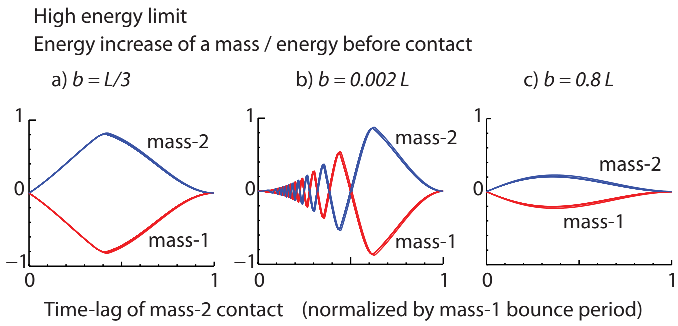

Figure S1: **Large energy limit for passive bouncing.** We show the relative energy transfer for the large energy limit, when gravity is not an issue, nor are many other parameters. a) $b = L/3$. b) The two masses very close together $b = 0.002L$. The two masses go through many oscillations before both of them go to flight phase. c) The two masses far apart. There is little interaction. When $b \approx L$, the relative energy transfer goes to zero. d) When $m_2 = 0.001m_1$, $b = L/3$ and initial drop heights ($H_1 = H_2$, not energies) are equal, the relative change in energy of m_1 is essentially zero, but the relative change in the energy of m_2 can be up to a factor of 3.

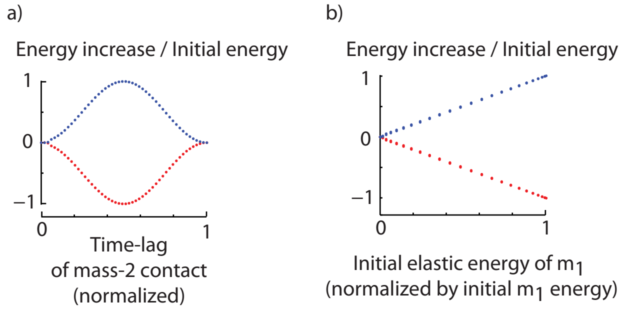

Figure S2: **Masses very close together during passive bouncing.** When the two masses are very close together, the mass in contact leaves contact as soon as the second mass makes contact. Thus the entire elastic energy of mass-1 is lost by mass-1, and transferred to mass-2. The two figures show energy transfer at the point when one of the two masses takes off.

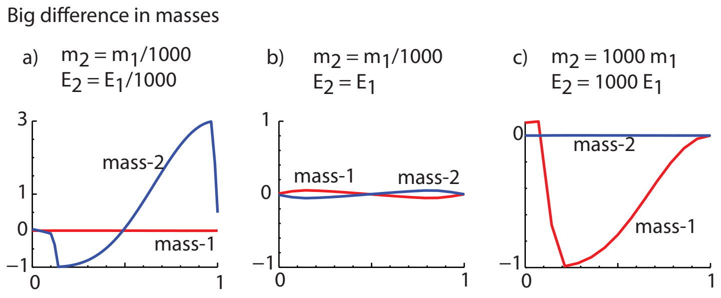

Figure S3: **Very different masses and passive bouncing**. a-b) Lighter mass m_2 lands second. a) Dropped from the same height. b) Dropping with similar initial energies (different heights). c) Heavier mass m_2 lands second.

References

[1] Pattar U, Joshi AW (2002) Head-on collision of two balls revisited. Resonance 7: 67-77.

[2] Cross R (2007) Vertical bounce of two vertically aligned balls. American Journal of Physics 75: 1009-1016.
